# Effect of FOLFOX on minimal residual disease in Stage III colon cancer and risk of relapse

**DOI:** 10.3332/ecancer.2019.935

**Published:** 2019-06-27

**Authors:** Nigel P Murray, Sócrates Aedo, Ricardo Villalon, Marco Antonio López, Simona Minzer, Lorena Muñoz, Shenda Orrego, Luis Contreras, Lucas Arzeno, Eghon Guzman

**Affiliations:** 1 Servicio de Medicina, Hospital de Carabineros de Chile, Simón Bolívar 2200, Ñuñoa, Santiago 7770199, Chile; 2 CTC Unit, Faculty of Medicine, University Finis Terrae, Providencia, Santiago 7501015, Chile; 3 Faculty of Medicine, University Finis Terrae, Providencia, Santiago 7501015, Chile; 4 Servicio de Coloproctologia, Hospital de Carabineros, Simón Bolívar 2200, Ñuñoa, Santiago 7770199, Chile; 5 Faculty of Medicine, University Mayor, San Pio X 2422, Providencia, Santiago 7500041, Chile

**Keywords:** colon cancer, circulating tumour cells, micro-metastasis, minimal residual disease, prognosis

## Abstract

**Introduction:**

25% of Stage III colon cancer patients relapse within 5 years due to minimal residual disease (MRD) not eliminated by surgery and chemotherapy. We hypothesise that sub-types of MRD, defined by circulating tumour cells (CTCs) and bone marrow micro-metastasis (mM) have different types and kinetics of relapse.

**Patients and Methods:**

One month of curative surgery and 1 month after completing six cycles of FOLFOX chemotherapy blood and bone marrow samples were taken to detect CTCs and mM using immunocytochemistry with anti-carcino-embryonic antigen (CEA). Follow up was up to 5 years or disease progression defined as new images on CT scanning. Survival curves using Kaplan–Meier (KM) and Restricted Mean Survival Time (RMST) were calculated for three prognostic groups: CTC and mM negative, CTC negative mM positive, and CTC positive.

**Results:**

76 patients (39 men) participated, mean age 67 years, median follow-up 3.6 years. The response to chemotherapy was heterogeneous and MRD pre-treatment did not predict response to therapy. Of 21 patients MRD (−), 20 remained MRD negative and one patient became mM (+); of 21 patients mM (+), 10 became MRD (−), 8 remained the same and 3 became CTC (+); of the 34 CTC positive, 8 became MRD (−), 8 with only mM, and 18 remained positive.

After chemotherapy, 38 patients were negative for CTC and mM, 17 were positive for only mM, and 21 for CTCs. For the whole cohort, the 5 year KM was 58%, the median survival was not reached. For the three prognostic groups, the KM 5-year survivals were 87%, 58%, and 4%, respectively, the median survival for patients MRD negative and mM only was not reached. RMST for the whole cohort was 3.6 years, for the three prognostic groups the RMST was 4.6 years, 4.0 years, and 1.5 years, respectively. Serum CEA was significantly higher pre-surgery in the CTC positive group. There were no significant differences with respect to age or sex between the three groups.

**Conclusions:**

MRD subtypes pre-chemotherapy did not predict treatment response. Post-chemotherapy MRD subtypes were associated with the pattern of failure and time to failure. MRD negative patients had an excellent prognosis with 87% disease-free survival at 5 years. Those with only mM had a similar outcome up to 2 years and then were at increasing risk of late failure. Patients who were CTC positive had a high risk of early failure. MRD subclassification may be useful to define the risk of relapse in Stage III colon cancer patients and warrants further studies with a larger number of patients.

## Introduction

Following surgery for Stage III colon cancer, the NCCN guidelines (2008) [1] recommend the use of adjuvant chemotherapy. This is because up to 30% of patients with Stage III colon cancer will develop disease recurrence within 5 years of surgery [2]. At the time of diagnosis and surgical resection, these patients had no signs of distant metastasis and as such, disease recurrence is a result of occult tumour dissemination prior to treatment. These disseminated tumour cells that remain after curative surgery are termed minimal residual disease (MRD). Circulating tumour cells (CTCs) detected after curative surgery are associated with decreased disease-free survival and overall survival [3]. Although bony metastases are infrequent in patients with colon cancer, the presence of disseminated tumour cells in bone marrow samples has been reported to be of the order of 30% and is associated with a worse prognosis [4]. These undetected micro-metastatic tumour cells are responsible for disease relapse, thus the detection of these cells in easily accessible tissues, such as blood and/or bone marrow, may be important to identify patients at risk of treatment failure and who could benefit from other therapeutic strategies [3].

We hypothesise that there are at least two sub-types of MRD, that those patients with CTCs have a worse prognosis and shorter times to treatment failure, while those with only bone marrow micro-metastasis are at risk of late failure. Tumour cells that are not actively proliferating are relatively resistant to chemotherapy; in addition, they may have phenotypic characteristics that confer resistance to anti-proliferative agents. Many tumour cells found in the bone marrow have been reported to quiescent and are not eradicated even with high-dose chemotherapy [5].

We present a prospective observational study of the effect of six cycles of FOLFOX chemotherapy on MRD in Stage III colon cancer patients and the association with time to relapse and progression-free survival.

## Patients and methods

A prospective single-institution study of consecutive patients who complied with the following criteria: newly diagnosed non-metastatic colon cancer, absence of previous colon cancer, or inflammatory colon disease and absence of metastasis seen on CT scan imaging of thorax, abdomen, and pelvis.

For each patient, after giving informed written consent, the following were recorded: a) sex, b) age, c) date of surgical treatment, and d) depth of primary tumour invasion (T) and nodal infiltration (N) according to the American Joint Committee on Cancer (AJCC) tumour/node/ metastasis (TNM) classification and staging system for colon cancer [6]. Serum levels of carcino-embryonic antigen (CEA) were taken the week before surgery and measured using the Cobas e601 analytical system (Roche Diagnostics, USA). A serum level of <3.0 ng/ml venous blood was considered to be normal. Patients classified as Stage III were included in the study.

### CTC detection

Blood samples were collected 1 month after curative surgery, defined as a surgical bed free of any gross residual tumour and surgical resection margins pathologically negative for tumour invasion, and 1 month after completing six cycles of FOLFOX chemotherapy.

Venous blood was collected using a 21G butterfly needle, the first 5 ml was discarded to prevent possible contamination by epithelial cells and the second 8 ml was collected into tubes containing ethylenediaminotetraacetic acid (EDTA, BD-Vacutainer®). The samples were transported at room temperature and processed within 24 hours.

Mononuclear cells were obtained using differential gel centrifugation with Histopaque 1.077® (Sigma-Aldrich, USA) according to the manufacturer’s instructions. The obtained cells were re-suspended in 100 μl of autologous plasma and 25 μl aliquots of cell suspension used to prepare four slides (silanised, DAKO, USA), they were air dried for 24 hours and finally fixed using a solution of 70% ethanol, 5% formaldehyde, and 25% phosphate buffered saline pH 7.4 (PBS) for 5 minutes and washed twice with PBS.

The slides were processed within 1 hour of fixation and incubated with monoclonal anti-CEA clone 11-7 (DAKO-USA) for 1 hour at room temperature, CTCs were identified using an alkaline-phosphatase-anti-alkaline phosphatase based system (LSAB2, DAKO, USA) with neofuschin as the chromogen and levisamole as an endogenous alkaline phosphatase inhibitor. Positive samples underwent a second process using anti-CD45 clone 2B11 + PD7/26 (DAKO, USA), incubated for 1 hour at room temperature and identified using a peroxidase-based system (LSAB2, DAKO, USA) with DAB (3,3ʹdiaminobenzadine tetrachloride) as the chromogen.

**Figure 1.**
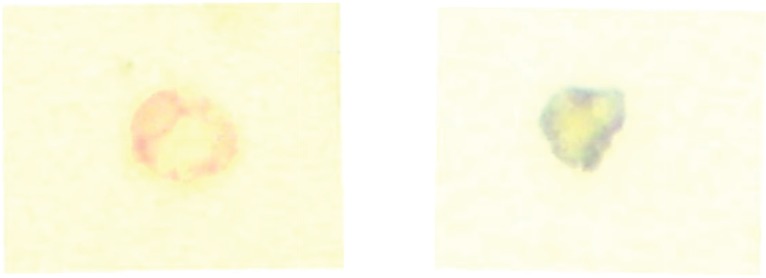
Circulating tumour cell and leukocyte and the expression of CEA. CTC expressing CEA (red) and negative for membrane CD45. Leukocyte negative for CEA and positive for membrane CD45.

A CTC was defined according to the morphological criteria of ISHAGE [7], as a nucleated cell expressing CEA but not CD45. A positive test was defined as the detection of at least one cell/8 ml venous blood (Figure 1).

### Bone marrow micro-metastasis

Although previous studies have used bone marrow aspirates to detect micro-metastasis, we used “touch preparations” from bone marrow biopsy specimens.

It has been reported that prostate tumour cells detected in bone marrow aspirates are phenotypically different from those prostate cells detected in bone marrow biopsies and may not represent “true” micro-metastasis but rather cells circulating in the bone marrow [8].

A bone marrow biopsy was taken from the posterior superior iliac crest 1 month after surgery and 1 month after completing chemotherapy. The samples were used to prepare four “touch preps” using silanised slides (DAKO, USA). The slides were processed in the same as for CTCs.

**Figure 2.**
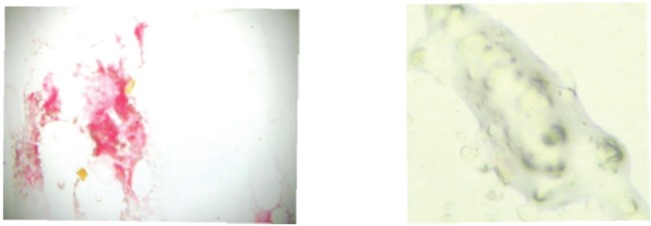
Bone marrow positive and negative for CEA expressing cells. Micro-metastasis CEA positive (red). Bone marrow negative for CEA.

After completing chemotherapy, the patients were divided into three prognostic groups: patients negative for both CTCs and micro-metastasis, patients negative for CTCs and positive for micro-metastasis, and third those CTC positive with or without the presence of micro-metastasis (Figure 2).

### Study end point

The primary study end point was the presence of metastasis detected on CT scan and the secondary end point the time from surgery to the detection of metastasis.

### Follow-up

Patients underwent three monthly follow-ups for the first 2 years then six monthly until 5 years of follow-up were completed. Relapse was defined as a new lesion detected on CT scanning of thorax, abdomen, and pelvis. Patients were censured at the time of relapse or after 5 years of follow-up.

### Statistical analysis

The analysis was performed using the program Stata (Stata/SE 15.0 for Windows®, Copyright 1985–2017 StataCorp LLC) using descriptive statistics for measurements of central tendency (mean and median) and dispersion [standard deviation (SD) and inter-quartile range (IQR)]. Nominal dichotomous variables were described as proportions with their respective confidence intervals [9]. The three prognostic groups determined after chemotherapy were compared for differences in age, sex, and serum CEA pre-surgery. Pearson’s Chi-squared was used to compare frequencies and the Kruskal–Wallis test to determine whether samples originated from the same distribution. A *p*-value of <0.05 was taken to signify statistical significance and all tests were two-tailed [9].

To evaluate the time to treatment failure, a non-parametric survival analysis was performed at three and 5 years of follow-up. The survival proportion and median survival for the three proportion prognostic groups were determined using the Kaplan–Meier model [9, 10]. The Restricted Mean Survival Time (RMST) for treatment failure was determined for 5 years of follow-up for the whole cohort and each prognostic group. The RMST establishes the expected time for an event to occur during a 5 year observation period and its value is the area under the Kaplan–Meier non-parametric survival curve [10]. The Log-Rank test was used to compare disease-free progression survival times for each prognostic group [10].

The analysis of disease-free progression survival did not fulfil the proportional risk criteria of the Cox regression model, and as such a flexible parametric survival model (FP) was used to predict the RMST and hazard ratios [11, 12]. According to the proposed hypothesis for the prediction of treatment failure, an FP model was built using the predictor variables of age, sex and prognostic group. The FP model is a regression method in which the dependent variable is the disease-free progression survival. The discrimination of the predictive model was determined using Harrell’s C discrimination index [13].

### Ethical considerations

The study was approved by the local ethics committee and fully complied with the Declaration of Helsinki.

## Results

The observed cohort included 76 patients, 39 (51%) males with a median age of 67 years (IQR 16 years) and a median follow up time of 3.6 years (range 0.25–5.0 years). Patients negative for both CTC and micro-metastasis had a significantly higher frequency of well-differentiated tumours and significantly lower frequency of lymphatic and vascular invasion in the primary tumour. There were no significant differences with respect to age, sex or peri-neural invasion. Overall, 42/76 (55%) of patients had an increased serum CEA; the frequency of an abnormal level increased from patients negative for both CTC and micro-metastasis to patients CTC positive (*p* = 0.03). With respect to the median serum CEA level, the only significant difference was between patients negative for both CTC and micro-metastasis and patients CTC positive (Table 1).

### Effect of chemotherapy on minimal residual disease

Prior to chemotherapy, 21 (28%) patients were negative for both CTC and micro-metastasis, 21 (28%) positive for micro-metastasis and negative for CTCs, and 34 (44%) were CTC positive. The response to chemotherapy was heterogeneous and the type of minimal residual disease pre-chemotherapy did not predict the response (Table 2).

### Minimal residual disease after chemotherapy and patient outcome

**Figure 3.**
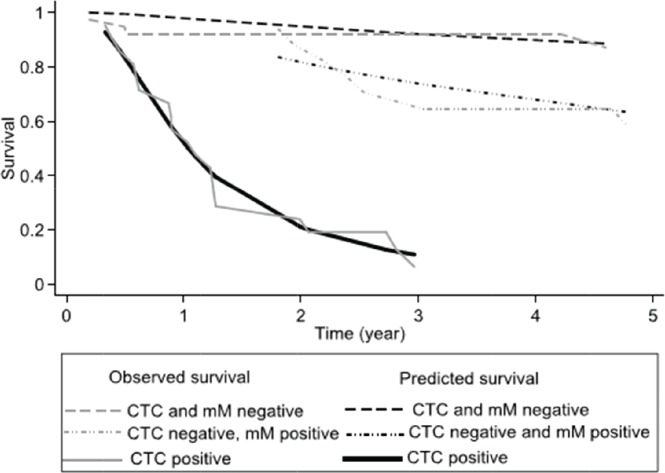
Comparing the observed survival (Kaplan–Meier) and predicted survival (flexible parameter model) at 5 years according to prognostic group.

38 patients (50%) were both CTC and micro-metastasis negative, 17 (22%) patients were only positive for micro-metastasis, and finally, 21 (28%) were CTC positive. 31 (41%) patients relapsed within the 5-year study period. After 3- and 5 years of follow up, the Kaplan–Meier survival for relapse-free survival was, respectively, 64% (95% CI 52%–74%) and 58% (95% CI 46%–69%), the median relapse-free survival was not reached. For each prognostic group, the three and five relapse-free survivals are shown in Table 3. Individuals negative for both CTCs and micro-metastasis and those with only micro-metastasis did not reach the median relapse-free survival. Differing those patients with CTCs showed a median relapse-free survival of 1.1 years (95% CI 0.6–1.28 years). Figure 3 shows the results as a graph.

The RMST for treatment failure for the whole cohort was 3.6 years (95% CI 3.2–4.0 years). Table 4 shows the RMST at 5 years for each prognostic group, from the observed Kaplan–Meier and Predicted (FP model) survival models. The Log-rank test showed a significant difference between groups (*p* < 0.01). There was no significant difference for sex or age of the patient (*p* = 0.99). The calculated hazard ratios (baseline CTC and micro-metastasis negative) were for CTC negative micro-metastasis positive HR 1.30 (*p* = 0.02) and CTC positive 3.35 (*p* > 0.01). The observed survival (Kaplan–Meier) was concordant to the survival predicted by the flexible parameter model, Harrell’s C was 0.82 classified as good.

## Discussion

In Stage III colon cancer treated with curative surgery and adjuvant FOLFOX chemotherapy, the relapse rate is reported to be approximately 30%. In this small study, a group of 76 patients 40% relapsed within the 5-year study period. The inference is that undetected minimal residual disease not eliminated by chemotherapy was present and was responsible for the relapse of the patient.

Although all of the patients were classified as Stage II, the individual behaviour of cancer is heterogeneous with differing responses to the same chemotherapy regime, which in turn leads to variable outcomes.

In the study, we used a Histopaque® differential centrifugation method to enrich CTCs from whole blood and then standard immunocytochemistry to detect CTCs. Detection of CTCs is method dependent, CTCs are a rare event being present in very low concentrations in the blood and all of the detection methods have an enrichment method. The only FDA approved system, CellSearch® is an EpCAM based method, although EpCAM is a conventional marker expressed by cancer cells of epithelial origin it is not expressed by all CTCs. CTCs which have undergone the epithelial-mesenchymal-transition have decreased expression of epithelial markers, such as EpCAM and cytokeratins [14], and will not be detected. EpCAM positive cells have been detected in patients with benign inflammatory disease and adenomatous polyps [15] leading to false-positive results. Whether intestinal mucositis post-chemotherapy is associated with circulating epithelial cells is an unknown parameter. As such EpCAM cannot be considered as a universal marker for CTC detection.

Non-specific enrichment methods rely on cell size, cell density, and deformability. Density gradient centrifugation is a conventional approach for separating blood components on the basis of differing sedimentation coefficients, with a reported recovery rate of spiked cells of 80%–87% [16]. Although inexpensive and reliable the disadvantages include the loss of large CTCs and cell clusters and the low purity due to the presence of leukocytes. Membrane microfilters have been developed which allow the rapid processing of large blood volumes but again have the same disadvantages as differential centrifugation. The use of microfluidics permits excellent purity with high capture rates but requires pre-processing to reduce the volume of the sample. The use of non-EpCAM-based approaches for CTC enrichment has been reviewed by Tellez *et al* [17]. After enrichment we used anti-CEA immunocytochemistry to identify CTCs, we acknowledge that immunocytochemistry is less sensitive by a factor of 10–100 fold than RT-PCR but can be implemented in the routine laboratory of a general hospital. We suggest that the sensitivity may not be an important factor; the question is the clinical utility. As yet, independent of the method to detect CTCs, no cut-off for a lower limit of detection has been established to determine clinical utility. The importance of this observation is the following; it may not be necessary to eliminate all CTCs to obtain a clinically important effect. This has been seen in haematological malignancies, patients transplanted for chronic myeloid leukaemia may have been in remission clinically and according to standard haematological parameters for years, yet analysis using RT-PCR demonstrates the presence of very low numbers of leukemic cells in the bone marrow and the patients had remained relapse free for more than ten years [18, 19]. In the study presented, there was a clinically useful association in that patients with a high risk of early relapse were identified. Thus, the lower sensitivity of Ficoll gel density gradient may be sufficient to detect CTCs at levels that are clinically significant, but it was beyond the scope of this study to answer this question.

More recently the use of circulating free DNA has been used as a prognostic marker. In colon cancer-specific DNA mutations have been identified, BRAF, K-ras, N-ras, and PIK3CA mutations. Circulating DNA is found in all people, as a result of necrosis, apoptosis, or secretion. Of this total circulating free DNA, a small percentage of free DNA can be identified in patients with cancer when there are specific mutations which separate ‘normal’ DNA from tumour ‘DNA’. The presence of circulating free tumour DNA for the mutations BRAF, K-ras, N-ras, and PIK3CA mutations has been used to identify patients with MRD and also as a marker for the resistance to EGFR therapy, such as cetuximab or panitumumab [20]. The use of CTC single cell DNA analysis compared with ctDNA detection showed a concordance of 97% but there was considerable heterogeneity of EFGR expression and genetic alterations in K-ras and PIK3CA [20]. This heterogeneity is seen both intra- and inter-patient, may explain the variable response rates to anti-EGFR therapy [21] and may change with time as a result of treatment, these differences are seen earlier in CTC single cell DNA analysis than ctDNA [22].

The CTC positive group was not divided into those micro-metastasis positive and negative, theoretically patients con be CTC positive and bone-marrow micro-metastasis negative, CTC dissemination occurring from a non-bone marrow micro-metastasis. This has been reported in other cancers, such as prostate [23] and breast [24]; however, in this reported study, only one patient was CTC positive and bone marrow micro-metastasis negative. In prostate and breast cancer studies, it has been reported that CTC positivity is associated with early failure independent of bone marrow micro-metastasis [23, 24]. In the clinical and for the single patient, all CTC positive patients were included as a single group.

The results of the study suggest that the presence of minimal residual disease after chemotherapy has clinically important consequences. The three sub-types of MRD pre-chemotherapy did not predict the results of treatment. Overall, 30 (39.9%) of patients had a different sub-type of MRD after chemotherapy when compared to the sub-type of MRD pre-chemotherapy. In patients converting from negative pre-chemotherapy to positive after chemotherapy for the presence of CTCs and/or micro-metastasis, a simplistic explication would be due to sampling error and that the limits of the methodology did not detect all of the patients with CTCs and/or micro-metastasis pre-chemotherapy. However, another explication is that a sub-group of tumour cells are relatively resistant to chemotherapy, a possible reason is the low proliferative index of these cells [5, 25]. In addition, the phenotypic characteristics of the tumour cells will determine the response to chemotherapy. CTCs positive for CD133 [26], CD26 [27] or increased thymidylate synthase expression [28] had poorer responses to chemotherapy and may continue to proliferate during treatment. There is little published evidence on the impact of pre and post-chemotherapy MRD in non-metastatic colon cancer. Romiti *et al* [29] reported using an EpCAM based system that in Stage III patients pre-chemotherapy CTC counts were not associated with prognosis, whereas post-chemotherapy CTC counts were. Post-operative CTCs but not pre-operative CTCs are associated with a worse prognosis [30] as are CTCs post FOLFOX for Stage III cancer [31].

In our reported study, the three sub-types of MRD were associated with differing clinical outcomes. Patients MRD negative had an excellent 5-year disease-free survival of 87% with a mean time to failure of 4.6 years. Those patients positive for CTCs had a much worse prognosis with a 5-year disease-free survival of only 6% and a mean time to progression of 1.5 years. Thus patients negative for CTCs but positive for micro-metastasis had a similar outcome to MRD negative patients for the first 18 months post-chemotherapy. Thereafter, there was an increasing relapse rate, with a disease-free progression after 5 years of 62% and a mean time to relapse of 4.0 years. The results suggest that these patients are at risk of late relapse.

Our results suggest that there are at least three sub-types of MRD, each with a differing pattern of clinical relapse and time to failure. Patients MRD negative after chemotherapy have an excellent prognosis, while those who are CTC positive have a poor prognosis and may benefit from additional therapies. Importantly, there is a group of patients who are CTC negative but with evidence of MRD who are at risk of late relapse. Interesting in patients with non-metastatic breast [32] and non-metastatic prostate cancer [23], similar patterns of relapse have been reported.

We recognise that the small number of patients limits the conclusions; however, the results suggest that the sub-classification of MRD gives meaningful clinical results and warrants larger multi-centre studies.

## Conclusions

The results of the study suggest that the sub-type of MRD detected after chemotherapy may have clinical meaningful utility in the risk classification of patients for relapse. MRD status pre-chemotherapy does not predict the outcome of treatment, with there being a heterogeneous response. The results warrant further larger scale studies.

## Funding

The research was funded by a Hospital de Carabineros de Chile Research Grant.

## Conflict of interest

Dr Murray has received consultancy fees from ViatarCTC Solutions, Boston, USA.

## Figures and Tables

**Table 1.** Clinical-pathological characteristics according to prognostic group.

Characteristic	CTC + mM negative *N* = 38	CTC negative mM Positive *N* = 17	CTC positive *N* = 21	*p* value two tail
Age (years) Median; (IQR)	66 (16)	68 (15)	68 (19)	0.62^a^
Male sex N (%)	17 (44)	8 (47)	14 (67)	0.25^b^
Tumour differentiation Well Moderate Poor Lymphatic invasion Vascular invasion Peri-neural invasion	22 12 4 8 8 2	7 8 2 4 7 18	4 8 9 13 18 3	0.01^b^ 0.004^b^ <0.001^b^ 0.44^b^
CEA % increased *n* (%) median (IQR) ng/ml	16 (42) 2.96 (IQR 2.05–5.30)	7 (41) 3.05 (IQR 2.22–8.03)	16 (71) 7.83 (IQR 2.49–17.08)	0.03^b^ Gp A versus GpC 0.002^a^

IQR = inter quartile range; CTC = circulating tumour cell; mM = micro-metastasis.

a Krusal–Wallis Test.

b Chi squared.

Of the CTC group, only 1/21 (5%) patient was negative for micro-metastasis.

**Table 2.** Minimal residual disease pre-chemotherapy and post-chemotherapy.

Pre-chemotherapy	Post-chemotherapy
CTC and mM (−) *N* = 21	CTC and mM (−) *N* = 20
	CTC (−) and mM (+) *N* = 1
	CTC (+) *N* = 0
CTC (-) and mM (+) *N* = 21	CTC and mM (−) *N* = 10
	CTC (−) and mM (+) *N* = 8
	CTC (+) *N* = 3
CTC (+) *N* = 34	CTC and mM (−) *N* = 8
	CTC (−) and mM (+) *N* = 8
	CTC (+) *N* = 18

**Table 3.** Comparing observed survival (Kaplan Meier) versus predicted survival (Model FP) for treatment failure at 3 and 5 years.

	% Observed survival 3 years (95% CI)	% Predicted survival 3 years	% Observed survival 5 years (95% CI)	% Predicted survival 5 years
CTC and mM negative	92% (77%–97%)	92%	87% (67%–95%)	88%
CTC negative mM positive	71% (43%–87%)	74%	58% (32%–76%)	62%
CTC positive	6% (0.5%–24%)	11%	6% (0.5%–24%)	4%

CTC = circulating tumour cell; mM = micro-metastasis

**Table 4.** RMST at 5 years for treatment failure according to prognostic group.

	RMST Kaplan–Meier years (95% CI)	RMST FP model
CTC and mM negative *N* = 38	4.6 years (4.2–5.0 years)	4.7 years
CTC negative and mM positive *N* = 17	4.0 years (3.4–4.7 years)	4.0 years
CTC positive	1.5 years (1.0–2.0 years)	1.4 years

CTC = circulating tumour cells; mM = micrometastasis, RMST = restricted mean survival time; FP = flexible parameter.
